# Molecular Confirmation of Ranavirus Infection in Amphibians From Chad, Africa

**DOI:** 10.3389/fvets.2021.733939

**Published:** 2021-09-16

**Authors:** Erin K. Box, Christopher A. Cleveland, Kuttichantran Subramaniam, Thomas B. Waltzek, Michael J. Yabsley

**Affiliations:** ^1^Southeastern Cooperative Wildlife Disease Study, Department of Population Health, College of Veterinary Medicine, University of Georgia, Athens, GA, United States; ^2^Department of Infectious Diseases and Immunology, College of Veterinary Medicine, University of Florida, Gainesville, FL, United States; ^3^Emerging Pathogens Institute, University of Florida, Gainesville, FL, United States; ^4^Warnell School of Forestry and Natural Resources, University of Georgia, Athens, GA, United States

**Keywords:** ranavirus, amphibian, reptile, phylogeography, Africa

## Abstract

Ranaviruses are DNA viruses (Family *Iridoviridae*; Subfamily *Alphairidovirinae*) and ranaviral disease is considered an emerging infectious disease of ectothermic vertebrates. Ranavirus infection can have varying pathological effects on infected amphibians, reptiles, and fish, most notably causing significant mortality events and population declines. Despite having a broad global range with reports from six continents, only a single incidental finding in *Xenopus longipes* from mainland Africa (Cameroon) is known and lacks molecular confirmation. Thus, there is a considerable knowledge gap concerning ranaviruses in Africa. We opportunistically obtained tissue samples from 160 amphibians representing five genera (*Hoplobatrachus, Hylarana, Ptychadena, Pyxicephalus*, and *Xenopus*) and two turtles (*Pelomedusa* sp.) from Chad, Africa. Samples were tested for ranavirus infection using a conventional PCR assay targeting the major capsid protein (MCP). A total of 25/160 (16%) frogs tested positive including 15/87 (17%) *Hoplobatrachus occipitalis*, 10/58 (17%) *Ptychadena* spp., 0/3 *Pyxicephalus* spp., 0/9 *Xenopus* spp., and 0/3 *Hylarana* spp. One of two turtles tested positive. Partial MCP gene sequences indicated all samples were >98% similar to several frog virus 3 (FV3)-like sequences. Additional gene targets (DNA polymerase [DNApol], ribonucleotide reductase alpha [RNR- α], ribonucleotide reductase beta subunit [RNR- β]) were sequenced to provide further detailed classification of the virus. Sequences of individual gene targets indicate that the ranavirus detected in frogs in Chad is most similar to tiger frog virus (TFV), a FV3-like virus previously isolated from diseased amphibians cultured in China and Thailand. Full genome sequencing of one sample indicates that the Chad frog virus (CFV) is a well-supported sister group to the TFVs previously determined from Asia. This work represents the first molecular confirmation of ranaviruses from Africa and is a first step in comparing ranavirus phylogeography on a local and global scale.

## Introduction

Members of the genus *Ranavirus* are double-stranded DNA viruses (Family *Iridoviridae*; Subfamily *Alphairidovirinae*) that can infect fish, amphibians, and reptiles (1). Transmission of some ranaviruses can occur between hosts of these taxonomic classes (2). Although asymptomatic infections may occur, in recent decades it has been recognized that ranaviruses can cause epizootics (3, 4). Ranaviruses induce systemic infections with variable presentation of disease (5). Clinical signs in amphibians include buoyancy problems, anorexia, swelling of the legs and body, redness, hemorrhages, cutaneous erosions, and ulcerations (6). In reptiles, common clinical signs include swelling of the head and extremities, skin ulcerations, and ocular discharge (5).

*Ranavirus* infection has been reported on every continent except Antarctica and is cited as a cause of amphibian, reptile, and fish die-offs (6–8). Epizootic hematopoietic necrosis virus (EHNV) in fish and ranaviruses in amphibians are reportable to the Office International des Epizooties (OIE) (7, 9, 10). Although some new reports are due to enhanced surveillance and testing, it is likely that both host and geographic range of infection is also increasing (7). The introduction of ranavirus into new regions may be due to numerous factors, including the exotic pet trade, commercialization of amphibians as food, and the import of animals for research (11).

As amphibian populations worldwide experience increasing threats to their stability, it is important that causes of mortality events are thoroughly investigated. To date, there is a single report of ranavirus from mainland Africa (Cameroon), and it was an incidental finding in *Xenopus longipes* that lacked molecular confirmation (12). Ranavirus has also been detected *via* qPCR in amphibians in Madagascar, although results were not confirmed with sequence analysis (13). Because of the high diversity of fish, amphibian, and reptile species in Africa, it is important to assess the distribution and host range of ranaviruses in Africa.

The aim of this study was to utilize opportunistically collected frog and turtle tissue samples to screen for the presence of ranavirus in Chadian amphibian populations. Assessing the ranavirus infection status of Chadian amphibian populations will aid in developing a better understanding of ranavirus phylogeography on a local and global scale. In doing this, we aim to inform existing knowledge gaps concerning the presence of ranaviruses among amphibian populations in Africa.

## Materials and Methods

### Tissue Collection

**Figure 1:**
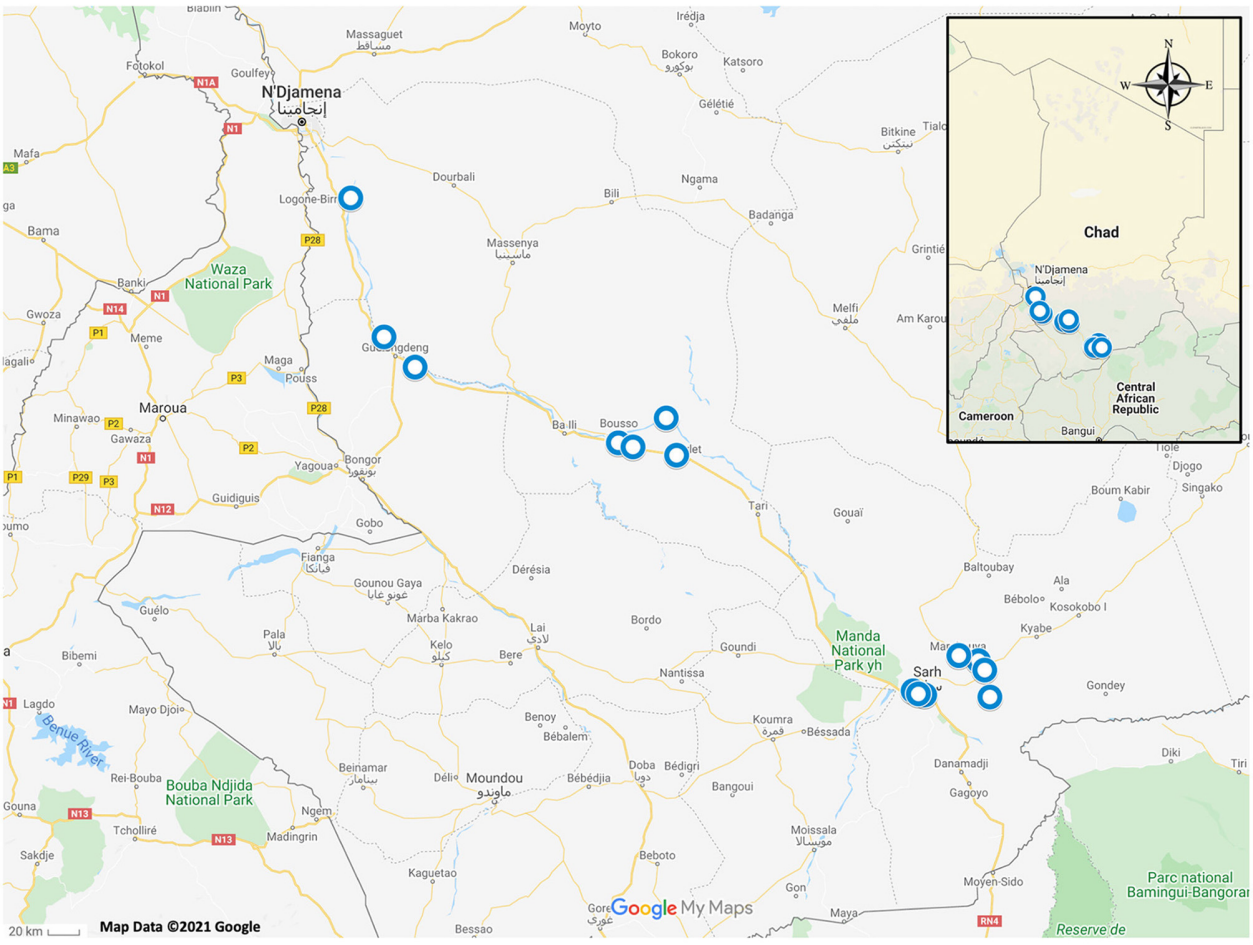
Map of Chad, Africa with blue circles depicting sampling locations of amphibians and reptiles which were included in this study.

Tissue samples were opportunistically obtained from animals captured for an unrelated study being conducted in several fishing villages along the Chari River Basin in Chad, Africa between June 2016 and July 2018 (Figure 1) (14). Local villagers obtained frogs either by hand or through the use of submersible nets baited with fish tissue (14). Two African helmeted turtles (*Pelomedusa* sp.) were incidentally collected and included in this study (14). Captured animals had no apparent clinical signs of disease upon routine observation by C. A. C. Animals were euthanized following American Veterinary Medical Association guidelines (15) and tissue samples (<1 cm toe clips) were acquired from each animal and preserved in 70% ethanol. All animal procedures were reviewed and approved by the University of Georgia's Institutional Animal Care and Use Committee (protocol no. A2016 07–024).

### DNA Extraction and PCR

DNA was extracted from tissue samples using a commercial DNA extraction kit (DNeasy Blood and Tissue Extraction Kit, QIAGEN, Germantown, MD). Samples were screened for ranavirus using conventional and real-time polymerase chain reaction (PCR). Initial determination of ranavirus infection status was done using conventional PCR primers targeting a ~500bp region of the ranavirus major capsid protein (MCP) gene (Table 1). Amplicons were purified from a 0.8% agarose gel stained with gel red (Biotium Inc., Hayward, California, USA) using a commercial gel-purification kit (QIAGEN). Bi-directional Sanger sequencing of amplicons was conducted by Georgia Genomics and Bioinformatics Core (Athens, GA) or GeneWiz (South Plainfield, New Jersey). The sequencing reads were edited and assembled in Geneious 10.2.6 software (Biomatters Limited, Auckland, New Zealand). The consensus sequences were then used as queries for BLASTN searches against the National Center for Biotechnology Information (NCBI) GenBank non-redundant (nr) nucleotide sequence database. Additional PCR analyses were performed targeting DNA polymerase (DNApol), ribonucleotide reductase alpha subunit (RNR-α) and ribonucleotide reductase beta subunit (RNR-β) gene targets (Table 1) of a select number of samples to determine genetic diversity of ranaviruses in Chad by geographic location and host species.

**Table 1:** Primers used for molecular diagnosis and differentiation of ranavirus from amphibian samples in Chad, Africa.

**Target Gene**	**Primer Name**	**Nucleotide Sequence (5′−3′)**	**Reference**
major capsid protein (MCP)	MCP 4R	GACTTGGCCACTTATGAC	(16)
	MCP 5	GTCTCTGGAGAAGAAGAA	
DNA polymerase (DNApol)	DNApol-F	GTGTAYCAGTGGTTTTGCGAC	(17)
	DNApol-R	TCGTCTCCGGGYCTGTCTTT	
ribonucleotide reductase alpha subunit (RNR-α)	RNR-AF	CTGCCCATCTCKTGCTTTCT	(18)
	RNR-AR	CTGGCCCASCCCATKGCGCCCA	
ribonucleotide reductase beta subunit (RNR-β)	RNR-BF	AGGTGTRCCRGGGYCGTA	(18)
	RNR-BR	GACGCTCCAYTCGACCACTT	
major capsid protein (MCP)	rtMCP-for	ACACCACCGCCCAAAAGTAC	(19)
	rtMCP-rev	CCGTTCATGATGCGGATAATG	
	MCP FAM Probe	6FAM CCTCATCGTTCTGGCCATCAACCA BHQ1	

### Real-Time PCR

Real-time PCR was performed to determine which samples had the highest viral DNA concentration and would be best for full viral genome sequencing. Real-time primers targeted the MCP gene and a FAM fluorescent probe was used for detection of the amplified product (Table 1). All samples were run in duplicate. The DNA sample with the lowest cycle threshold (Ct) value (A-21) was used for full ranaviral genome sequencing.

### Complete Genome Sequencing and Genome Annotation

The DNA extracted from a crowned bullfrog (*Hoplobatrachus occipitalis*; sample A-21) was used to prepare a DNA sequencing library with a NEBNext^®^ Ultra™ II DNA Library Prep Kit according to the manufacturer's instruction. Sequencing was performed on an Illumina MiSeq sequencer using a v3 chemistry 600-cycle kit. *De novo* assembly of the paired-end reads was performed using SPAdes 3.10.1 (20). The quality of the genome assembly was verified by mapping the reads back to the consensus sequence in Bowtie 2 and visually inspecting the alignments in Tablet (21, 22). The genome was annotated using the Genome Annotation Transfer Utility with the tiger frog virus isolate D2008 (TFV-D2008; GenBank Accession No. MT512502.1) used as the reference genome (23). Additional putative open reading frames (ORFs) were identified or removed by comparison to FV3 and TFV genome annotations (24, 25). The functions of the open reading frames (ORFs) were predicted based on BLASTP searches against the NCBI GenBank nr protein sequence database.

### Phylogenetic and Genetic Analyses

Supplementary Table 1 summarizes the ranaviruses, genes, and corresponding NCBI GenBank accession numbers used to generate five datasets for the phylogenetic and genetic analyses. Dataset 1 (DS1) contained the Chad ranavirus genome (sample A-21) and 44 fully sequenced ranavirus genomes retrieved from NCBI GenBank. These sequences were aligned using Mauve 2.4 software to visualize genomic inversions and obtain the locally collinear block (LCB) alignments (26). The LCB alignments were then concatenated in Geneious 10.2.6 and contained 144,720 nucleotide (nt) characters (including gaps) (27). MEGA X was used to determine best-fit models and perform Maximum Likelihood (ML) phylogenetic analyses (28).

To elucidate the relationship of Chad ranaviruses to other ranaviruses for which the full genome sequences have not been determined (e.g., the blood phyton ranavirus [BPRV], Dopasia gracilis ranavirus [DGRV], and Wamena virus [WV]), additional phylogenetic and genetic analyses were performed using four conserved genes encoding MCP, RNR-α, RNR-β, and DNApol. These gene alignments included partial sequences and missing data were coded as questions marks (?). Dataset 2 (DS2) contained 1,392 nt characters (including gaps) and consisted of seven partial (DGRV, BPRV, Chad ranavirus A-62, Chad ranavirus A-61, Chad ranavirus T17-01, Chad ranavirus 033c, and the consensus sequence of 18 identical Chad ranaviruses [i.e., A-19, A-20, A-21, A-22, A-24, A-25, A-27, A-35, A-50, A-51, A-54, A-58, A-60, A-64, A-73, A17-07, A17-08, 053]) and 45 complete MCP sequences. Dataset 3 (DS3) contained 3,042 nt characters (including gaps) and consisted of four partial (DGRV, BPRV, WV, and the consensus sequence of nine identical Chad ranaviruses [i.e., A-21, A-24, A-27, A-35, A-36, A-50, A-55, A17-07, 053]) and 44 complete DNApol sequences. Dataset 4 (DS4) contained 1,698 nt characters (including gaps) and consisted of three partial (DGRV, BPRV, and the consensus sequence of four identical Chad ranaviruses [i.e., A-21, A-50, A17-07, 053]) and 45 complete RNR- α ranavirus sequences. Dataset 5 (DS5) contained 1,164 nt characters (including gaps) and consisted of four partial (DGRV, BPRV, WV, and the consensus sequence of six identical Chad ranaviruses [i.e., A-21, A-27, A-36, A-50, A17-07, 053]) and 44 complete RNR- β sequences. ML phylogenetic analyses of DS2, DS3, DS4, and DS5 were performed as described above and genetic analyses were conducted using the Sequence Demarcation Tool Version 1.2 with the MAFFT option implemented (29).

## Results

### Prevalence Determined by PCR

Tissue samples from 160 frogs were tested, representing five genera (*Hoplobatrachus, Hylarana, Ptychadena, Pyxicephalus, Xenopus*). A total of 25/160 (16%) samples were confirmed positive for ranavirus using conventional PCR and Sanger sequencing. The prevalence for both *Hoplobatrachus occipitalis* (15/87) and *Ptychadena* spp. (10/58) was 17%. All three *Pyxicephalus* spp., nine *Xenopus* spp., and three *Hylarana* spp. were negative. Additionally, one of two (50%) African helmeted turtles was confirmed positive for ranavirus. Sequences from all MCP amplicons (~500 bp) were most similar (>98%) to several FV3-like virus sequences.

### Real-Time PCR

The lowest cycle threshold (Ct) value obtained from a real-time PCR sample was 17.94 from sample A-21, so this sample was used for full genomic sequencing and analysis.

### Complete Genome Sequencing and Genome Annotation

The *de novo* assembly of the 50,786,552 paired-end reads recovered a contiguous sequence of 106,120 bp. The G+C content of the genome was 54% with an average coverage of nine reads/nucleotides. The Chad ranavirus genome is predicted to encode 96 open reading frames (ORFs; Supplementary Table 2) and possesses a FV3-like ranavirus genome arrangement as observed among the members of the tiger frog virus (i.e., Chinese TFV and Thai ranaviruses) subclade (data not shown) (25, 30). Comparative genomic analyses revealed that the Chad ranavirus possesses a hypothetical protein (TFV ORF 28), which is only encoded by the Chinese TFV and Thai ranaviruses, but absent in all other ranaviruses. Chad ranavirus possesses a FV3 30R equivalent ORF (ORF 31 in Chad ranavirus; hypothetical protein), which is absent in the Chinese TFV and all Thai ranaviruses. Three hypothetical proteins (TFV ORFs 38, 43, 71), a L-protein-like protein (TFV 36), a thymidylate synthase (TFV 87), and an integrase-like protein (TFV 17) encoded by the Chinese TFV and all Thai ranaviruses were absent in the Chad ranavirus. TFV ORFs 6.5 and 59, encoding hypothetical proteins, were present in the Chad ranavirus, Chinese TFV and all Thai ranaviruses except Oxyeleotris marmorata ranavirus (OMRV) and Asian grass frog ranavirus (AGFRV). An ORF encoding a putative nuclear calmodulin-binding protein (TFV ORF 54) is only present in the Chad ranavirus, Chinese TFV, OMRV, and AGFRV. TFV ORF 97, encoding a hypothetical protein, is present in the Chad ranavirus, Chinese TFV, and all Thai ranaviruses except AGFRV. TFV ORFs 26 and 63, encoding hypothetical proteins, were present in the Chinese TFV, all Thai ranaviruses except OMRV, and the Chad ranavirus. The complete genome of the Chad ranavirus has been deposited in GenBank under accession number MW727505.

### Phylogenetic and Genetic Analyses

**Figure 2:**
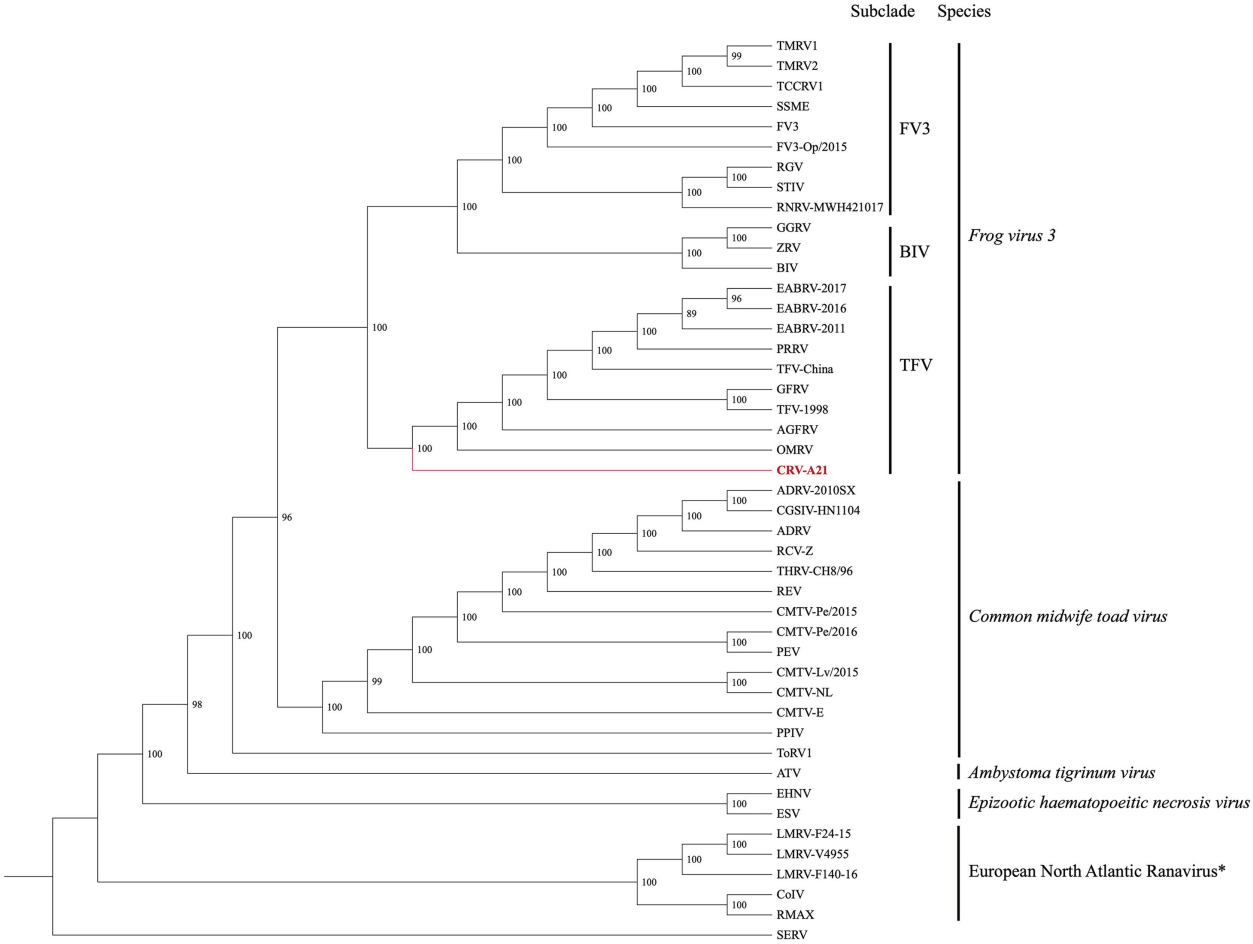
Cladogram illustrating the relationship of the newly sequenced Chad ranavirus (CRV-A21) to members of the genus *Ranavirus* (Family *Iridoviridae*; Subfamily *Alphairidovirinae*). The Maximum Likelihood (ML) phylogenetic analysis included the aligned genomes (concatenated locally collinear blocks) of 44 ranaviruses. Clade support was assessed by running 1,000 bootstrap replicates with values presented at each node. See Supplementary Table 1 for virus abbreviations. *Note: European North Atlantic Ranavirus has not been approved as a ranavirus species by the ICTV.

The Maximum Likelihood (ML) analysis based on the DS1 generated a well-supported tree with a topology similar to a recent analysis (Figure 2) (25). The ML tree supported the Chad ranavirus as the most basal branch of the TFV subclade within the larger FV3 clade. The ML analyses based on the DS2 demonstrated the monophyly of Chad ranaviruses within the TFV subclade (Supplementary Figure 1). The ML analyses based on the DS3, DS4, and DS5 supported the Chad ranaviruses and three squamate reptile ranavirus strains (BPRV, DGRV, and WV) as members of the TFV subclade (Supplementary Figures 2–4). Sequences from the MCP gene grouped separately from other ranaviruses, and all samples, except four, grouped together (Supplementary Figure 1). Sequences from the DNApol gene grouped together, as the sister clade to DGRV (Supplementary Figure 2). Analysis of the RNR- α and RNR- β genes produced similar topologies to the other genes (Supplementary Figures 3, 4).

Genetic analysis of the DS2 revealed the nucleotide identity of the Chad ranaviruses ranged from 98.5–99.8% when compared to each other, 97.5–98.5% when compared to members of the TFV subclade, and 94.0–99.3% when compared to other ranaviruses (Supplementary Table 3). Genetic analysis of the DS3 revealed the nucleotide identity of the Chad ranaviruses ranged from 98.4 to 99.6% when compared to other TFVs, and 95.9–98.9% when compared to other ranaviruses (Supplementary Table 4). Genetic analysis of the DS4 revealed the nucleotide identity of the Chad ranaviruses ranged from 99.1 to 99.6% when compared to other TFVs, and 94.6–99% when compared to other ranaviruses (Supplementary Table 5). Genetic analysis of the DS5 revealed the nucleotide identity of the Chad ranaviruses ranged from 98.5 to 99.2% when compared to other TFVs, and 96.1–98.6% when compared to other ranaviruses (Supplementary Table 6). Finally, the Mauve 2.4 analysis revealed that the Chad ranavirus genome possessed a FV3-like Ranaviral genome arrangement (Supplementary Figure 5) (30).

## Discussion

The objective of this study was to investigate the presence of ranavirus in the Chari River Basin of Chad, Africa using opportunistically collected tissue samples. Our results provide the first sequence confirmed detection of ranavirus in mainland Africa. Infections were detected in two anuran genera (*Hoplobatrachus* and *Ptychadena*) and one turtle species (*Pelomedusa* sp.) across the geographic range of our sampling efforts. Phylogenetic and genetic analyses, based on partial and complete genome datasets, supported the Chad frog ranaviruses as the sister group to the TFVs, described from Asian fish, amphibians, and reptiles [reviewed in Sriwanayos et al. (25)].

During this study, ranavirus was found in samples from crowned bullfrogs (*H. occipitalis*). Although they are referred to as bullfrogs, crowned bullfrogs are not closely related to other African, European, or North American bullfrog species (31). Generally, the *Hoplobatrachus* species (Family Dicroglossidae) are found throughout Southeast Asia; the crowned bullfrog is the only African member of the genus *Hoplobatrachus*, which are also referred to as tiger frogs (31). Tiger frog virus, the ranavirus strain with which our samples grouped most closely during analysis of the full genome, was first isolated in 2000 from *Hoplobatrachus tigerinus* (previously, *Rana tigrina*) in Chinese aquaculture facilities following severe mortality events (32, 33). Strains of TFV have also been associated with disease in cultured fish and frogs in Thailand (25). Interestingly, variation in presentation has been noted between some infected hosts in China and Thailand with some having cutaneous lesions and no internal gross lesions and others having classic internal lesions (e.g., enlargement of internal organs and petechial hemorrhages) (25). This contrasts with the lack of apparent disease in ranavirus-infected Chadian frogs, including *H. occipitalis*. Further surveillance is required to determine whether amphibian morbidity or mortality is being caused by ranaviruses in Chad and may have been missed in initial sampling, or whether these populations may be more resistant to ranaviral disease than Asian *Hoplobatrachus* species.

Samples from *Ptychadena* spp. also tested positive for ranavirus during this study. There is great species diversity within genus *Ptychadena* (Family Ptychadenidae) in Sub-Saharan Africa (34). Species are still being discovered and hotspots of endemism may be at risk if threatened with spreading range of ranavirus infections. Further testing of these species, especially in areas of high endemism may benefit conservation efforts and understanding of these amphibian populations.

Although no *Xenopus* tested positive for ranavirus in this study, the sample size was very small (nine individuals). It has been suggested that *Xenopus* may serve as viral reservoirs for ranaviruses, as asymptomatic individuals have been found to harbor low-level infections (35). Asymptomatic ranaviral infections have been detected in *Xenopus* sourced from multiple laboratory animal suppliers (35). Ranavirus has also been detected from feral, invasive *Xenopus* sampled in Chile during an amphibian health survey (36). These studies suggest that *Xenopus* may be resistant to clinical disease when infected with ranavirus and may contribute to expansion of ranavirus distribution. Whether this resistance to ranaviral disease exhibited by *Xenopus* is a consequence of coevolution with ranaviruses in Africa is yet unknown. It is also unknown whether this disease resistance occurs in wild *Xenopus* populations, as wild African *Xenopus* have not been widely surveyed for ranavirus infection or disease.

This detection of ranavirus in an African helmeted turtle, *Pelomedusa* sp., marked the first detection of ranavirus in a wild African reptile. Like all animals included in this study, this turtle showed no apparent clinical signs of disease upon sampling. This finding highlights the importance of wildlife health and disease monitoring, even when a positive result may be unexpected. Ranaviruses have been associated with disease in other species of aquatic turtles from Asia, Europe and North America (37–40) but there is also evidence that some species of aquatic turtles can have asymptomatic ranavirus infections (41, 42). As we detected only a single positive animal, and sampled only two, additional understanding of Chadian turtle populations and surveillance for ranavirus may elucidate what impact, if any, they have on these animals and animal populations.

Our analysis detected at least one ranavirus strain in the sampled individuals. It is also possible that the samples which grouped separately from the majority of our samples (033-C, T17-01, A-62, and A-62) during analysis of MCP gene sequences could represent additional species. Additional full-genome sequencing and analysis of these samples could lead to additional insight into relatedness of these ranavirus samples. We did not detect variation within the sequenced gene targets between the two ranavirus-positive amphibian species or across our sampling sites. Similarly, whole genome sequencing of the Chad ranavirus and subsequent phylogenomic analysis with other fully sequenced ranaviruses revealed a high similarity between Chad ranavirus and TFVs. The formation of a well-supported and distinct clade between Chad ranavirus and other TFVs, from two different continents, reveals that there is more diversity to this group of ranaviruses infecting a wide range of hosts.

It is possible that some of the animals screened with conventional PCR and considered negative during this study had ranavirus infections that were not detected, but may have been detected via other means, such as qPCR. It is also possible that viral load was considerably lower in the tissue type we tested (toe clips) and that virus may have been detected if tissues such as liver or kidney were sampled. Although this is a potential limitation of this study, samples were not acquired with ranavirus screening in mind. Future studies could be improved by targeting multiple tissue types which would aid in direct comparison with findings of other studies and create a standardized approach to ranavirus screening in wild animals.

Central African amphibian populations are greatly understudied (43). Frogs often play a significant role in local communities, highlighting a need for long-term studies in Central Africa to further understand frog population health (44). To local communities, frogs hold economic, medicinal, pest control, and nutritional value (44). The edible *Hoplobatrachus occipitalis* (one of the species positive for ranavirus) is extensively traded for food (44, 45). As amphibian trade is often cited as a factor in global ranavirus spread (6, 11), it is possible that amphibian trade could contribute to the spread of ranavirus on the African continent. A better understanding of the impact of ranaviruses on these amphibian populations may aid in conservation of these valuable amphibian populations.

Our results confirm the presence of ranavirus on mainland Africa. Genetic analysis of the Chad Ranavirus has allowed us to develop a better understanding of the global phylogeography of ranaviruses and is the beginning of an exploration of ranaviruses in Africa. Further investigation is necessary to better understand the geographic and host range of ranavirus on the continent, as well as how they may be impacting animal health.

## Data Availability Statement

The datasets presented in this study can be found in online repositories. The names of the repository/repositories and accession number(s) can be found in the article/Supplementary Material.

## Ethics Statement

The animal study was reviewed and approved by University of Georgia IACUC.

## Author Contributions

EB and CC compiled samples and performed testing and initial molecular analysis. KS and TW performed phylogenetic and genetic analyses. MY was the principal investigator for the experiment. All authors made substantial contribution to the conception, design, sample acquisition, analysis, and/or interpretation of the data for this study, agree to be personally accountable for their contributions, drafted, and reviewed the manuscript.

## Funding

Samples were collected during surveillance studies conducted in collaboration with the Carter Center in an effort to further the mission to eradicate Guinea worm disease. These efforts were made possible by financial and in-kind contributions from many donors to the Carter Center. A full listing of supporters is available at http://www.cartercenter.org/donate/corporate-government-foundation-partners/index.html. Additional support was provided by the wildlife management agencies of the Southeastern Cooperative Wildlife Disease Study member states through the Federal Aid to Wildlife Restoration Act (50 Stat. 917) and by a U.S. Department of the Interior Cooperative Agreement.

## Conflict of Interest

The authors declare that the research was conducted in the absence of any commercial or financial relationships that could be construed as a potential conflict of interest. The handling editor declared a past co-authorship with several of the authors TW and KS.

## Publisher's Note

All claims expressed in this article are solely those of the authors and do not necessarily represent those of their affiliated organizations, or those of the publisher, the editors and the reviewers. Any product that may be evaluated in this article, or claim that may be made by its manufacturer, is not guaranteed or endorsed by the publisher.
