# Supplementary material for: Molecular Confirmation of Ranavirus Infection in Amphibians From Chad, Africa
Source: Front Vet Sci. 2021 Sep 16;8:733939. doi: 10.3389/fvets.2021.733939 (PMC8481899; doi:10.3389/fvets.2021.733939)
Supplement: Supplementary file 1 [file Data_Sheet_1.docx]

Supplementary Material

**Table S1.** Ranavirus sequences used to construct the 5 datasets in the present study (Dataset 1; DS1, Dataset 2; DS2, Dataset 3; DS3, Dataset 4; DS4, and Dataset 5; DS5) and their GenBank accession numbers. NA: not available.

| **Virus name** | **Virus abbreviation** | **DS1** | **DS2** | **DS3** | **DS4** | **DS5** |
| --- | --- | --- | --- | --- | --- | --- |
| Ambystoma tigrinum virus | ATV | AY150217 | AY150217 | AY150217 | AY150217 | AY150217 |
| Andrias davidianus ranavirus | ADRV | KC865735 | KC865735 | KC865735 | KC865735 | KC865735 |
| Andrias davidianus ranavirus | ADRV-2010SX | KF033124 | KF033124 | KF033124 | KF033124 | KF033124 |
| Asian grass frog ranavirus | AGFRV | MT512497 | MT512497 | MT512497 | MT512497 | MT512497 |
| Bohle iridovirus | BIV | KX185156 | KX185156 | KX185156 | KX185156 | KX185156 |
| Chinese giant salamander iridovirus | CGSIV-HN1104 | KF512820 | KF512820 | KF512820 | KF512820 | KF512820 |
| Cod iridovirus | CoIV | KX574342 | KX574342 | KX574342 | KX574342 | KX574342 |
| Common midwife toad virus | CMTV-E | JQ231222 | JQ231222 | JQ231222 | JQ231222 | JQ231222 |
| Common midwife toad virus | CMTV-NL | KP056312 | KP056312 | KP056312 | KP056312 | KP056312 |
| Common midwife toad virus | CMTV-Lv/2015 | MF004272 | MF004272 | MF004272 | MF004272 | MF004272 |
| Common midwife toad virus | CMTV-Pe/2015 | MF125269 | MF125269 | MF125269 | MF125269 | MF125269 |
| Common midwife toad virus | CMTV-Pe/2016 | MF125270 | MF125270 | MF125270 | MF125270 | MF125270 |
| East Asian bullfrog ranavirus | EABRV-2011 | MT512498 | MT512498 | MT512498 | MT512498 | MT512498 |
| East Asian bullfrog ranavirus | EABRV-2016 | MT512499 | MT512499 | MT512499 | MT512499 | MT512499 |
| East Asian bullfrog ranavirus | EABRV-2017 | MT512500 | MT512500 | MT512500 | MT512500 | MT512500 |
| Epizootic haematopoietic necrosis virus | EHNV | FJ433873 | FJ433873 | FJ433873 | FJ433873 | FJ433873 |
| European catfish virus | ECV | KT989885 | KT989885 | KT989885 | KT989885 | KT989885 |
| European sheatfish virus | ESV | JQ724856 | JQ724856 | JQ724856 | JQ724856 | JQ724856 |
| Frog virus 3 | FV3 | AY548484 | AY548484 | AY548484 | AY548484 | AY548484 |
| Frog virus 3 | FV3-Op/2015 | MF360246 | MF360246 | MF360246 | MF360246 | MF360246 |
| Frog virus 3 isolate SSME | SSME | KJ175144 | KJ175144 | KJ175144 | KJ175144 | KJ175144 |
| German gecko ranavirus | GGRV | KP266742 | KP266742 | KP266742 | KP266742 | KP266742 |
| Goldfish ranavirus | GFRV | MT512501 | MT512501 | MT512501 | MT512501 | MT512501 |
| Lumpfish ranavirus isolate F140-16 | LMRV-F140-16 | MH665359 | MH665359 | MH665359 | MH665359 | MH665359 |
| Lumpfish ranavirus isolate F24-15 | LMRV-F24-15 | MH665358 | MH665358 | MH665358 | MH665358 | MH665358 |
| Lumpfish ranavirus isolate V4955 | LMRV-V4955 | MH665360 | MH665360 | MH665360 | MH665360 | MH665360 |
| Oxyeleotris marmorata ranavirus | OMRV | MT512502 | MT512502 | MT512502 | MT512502 | MT512502 |
| Pelophylax esculentus virus | PEV | MF538627 | MF538627 | MF538627 | MF538627 | MF538627 |
| Pike-perch iridovirus | PPIV | KX574341 | KX574341 | KX574341 | KX574341 | KX574341 |
| Poecilia reticulata ranavirus | PPRV | MT512503 | MT512503 | MT512503 | MT512503 | MT512503 |
| Rana catesbeiana virus isolate RC-Z | RCV-Z | MF187210 | MF187210 | MF187210 | MF187210 | MF187210 |
| Rana esculenta virus | REV | MF538628 | MF538628 | MF538628 | MF538628 | MF538628 |
| Rana grylio iridovirus | RGV | JQ654586 | JQ654586 | JQ654586 | JQ654586 | JQ654586 |
| Rana nigromaculata ranavirus isolate MWH421017 | RNRV-MWH421017 | MG791866 | MG791866 | MG791866 | MG791866 | MG791866 |
| Ranavirus maximus | Rmax | KX574343 | KX574343 | KX574343 | KX574343 | KX574343 |
| Short-finned eel ranavirus | SERV | KX353311 | KX353311 | KX353311 | KX353311 | KX353311 |
| Soft-shelled turtle iridovirus | STIV | EU627010 | EU627010 | EU627010 | EU627010 | EU627010 |
| Terrapene carolina carolina ranavirus | TCCRV | MG953518 | MG953518 | MG953518 | MG953518 | MG953518 |
| Testudo hermanni ranavirus | THRV-CH8/96 | KP266741 | KP266741 | KP266741 | KP266741 | KP266741 |
| Tiger frog virus | TFV-China | AF389451 | AF389451 | AF389451 | AF389451 | AF389451 |
| Tiger frog virus 1998 | TFV-1998 | MT512504 | MT512504 | MT512504 | MT512504 | MT512504 |
| Tortoise ranavirus isolate 1 | ToRV1 | KP266743 | KP266743 | KP266743 | KP266743 | KP266743 |
| Trioceros melleri ranavirus 1 | TMRV1 | MG953519 | MG953519 | MG953519 | MG953519 | MG953519 |
| Trioceros melleri ranavirus 2 | TMRV2 | MG953520 | MG953520 | MG953520 | MG953520 | MG953520 |
| Zoo ranavirus isolate 040414 | ZRV | MK227779 | MK227779 | MK227779 | MK227779 | MK227779 |
| Wamena virus | WV | NA | MT507284 | MT507284 | MT507284 | MT507284 |
| Blood python ranavirus | BPRV | NA | KM516715 | KM516724 | KM516733 | KM516742 |
| Dopasia gracilis ranavirus | DGRV | NA | KM516714 | KM516723 | KM516732 | KM516741 |
| Chad ranavirus A-19 | CRV A-19 | NA | MZ357042 | NA | NA | NA |
| Chad ranavirus A-20 | CRV A-20 | NA | MZ357042 | NA | NA | NA |
| Chad ranavirus A-21 | CRV-A21 | MW727505 | MZ357042 | MZ357049 | MZ357047 | MZ357048 |
| Chad ranavirus A-22 | CRV A-22 | NA | MZ357042 | NA | NA | NA |
| Chad ranavirus A-24 | CRV A-24 | NA | MZ357042 | NA | NA | MZ357048 |
| Chad ranavirus A-25 | CRV A-25 | NA | MZ357042 | NA | NA | NA |
| Chad ranavirus A-27 | CRV A-27 | NA | MZ357042 | NA | MZ357047 | MZ357048 |
| Chad ranavirus A-35 | CRV A-35 | NA | MZ357042 | NA | NA | MZ357048 |
| Chad ranavirus A-36 | CRV A-36 | NA | MZ357042 | NA | MZ357047 | MZ357048 |
| Chad ranavirus A-50 | CRV A-50 | NA | MZ357042 | MZ357049 | MZ357047 | MZ357048 |
| Chad ranavirus A-51 | CRV A-51 | NA | MZ357042 | NA | NA | NA |
| Chad ranavirus A-54 | CRV A-54 | NA | MZ357042 | NA | NA | NA |
| Chad ranavirus A-55 | CRV A-55 | NA | MZ357042 | NA | NA | MZ357048 |
| Chad ranavirus A-56 | CRV A-56 | NA | MZ357042 | NA | NA | NA |
| Chad ranavirus A-57 | CRV A-57 | NA | MZ357042 | NA | NA | NA |
| Chad ranavirus A-58 | CRV A-58 | NA | MZ357042 | NA | NA | NA |
| Chad ranavirus A-60 | CRV A-60 | NA | MZ357042 | NA | NA | NA |
| Chad ranavirus A-61 | CRV A-61 | NA | MZ357044 | NA | NA | NA |
| Chad ranavirus A-62 | CRV A-62 | NA | MZ357043 | NA | NA | NA |
| Chad ranavirus A-64 | CRV A-64 | NA | MZ357042 | NA | NA | NA |
| Chad ranavirus A-73 | CRV A-73 | NA | MZ357042 | NA | NA | NA |
| Chad ranavirus A17-07 | CRV A17-07 | NA | MZ357042 | MZ357049 | MZ357047 | MZ357048 |
| Chad ranavirus A17-08 | CRV A17-08 | NA | MZ357042 | NA | NA | NA |
| Chad ranavirus T17-01 | CRV T17-01 | NA | MZ357045 | NA | NA | NA |
| Chad ranavirus 033-C | CRV 033-C | NA | MZ357046 | NA | NA | NA |
| Chad ranavirus 53 | CRV 53 | NA | MZ357042 | MZ357049 | MZ357047 | MZ357048 |

| **Table S2.** Genome annotation of the Chad ranavirus (A-21) genome. | | | | | | | | | |
| --- | --- | --- | --- | --- | --- | --- | --- | --- | --- |
| ORF | Position (nt range) | | Product size (aa) | Predicted function and conserved domain | Best BLAST hit | | | | |
|  |  |  |  |  | Description | E-value | Accession no. | | |
| TFV 1 | 1-771 | | 256 | replication factor | putative replication factor [Pike perch iridovirus] | 0 | ANZ56919 | | |
| TFV 2 | 1485-2492 | | 335 | myristylated membrane protein | myristylated membrane protein [Chinese giant salamander iridovirus] | 0 | AGK44996 | | |
| TFV 3 | 2530-3369 | | 279 | hypothetical protein | hypothetical protein D1U33_gp003 [Common midwife toad virus] | 0 | YP_009508631 | | |
| TFV 4 | 3402-4616 | | 404 | hypothetical protein | Orf229L protein-like protein [Tiger frog virus] | 0 | QKG82163 | | |
| TFV 5 | 4654-4836 | | 60 | hypothetical protein | hypothetical protein [Tiger frog virus] | 4.98E-35 | QKG82677 | | |
| TFV 6 | 5277-5879 | | 200 | US22 family protein | putative US22 family protein [Tortoise ranavirus] | 5.34E-137 | AJR29236 | | |
| TFV 6.5 | 5872-6129 | | 85 | hypothetical protein | hypothetical protein D1R28_gp007 [Bohle iridovirus] | 1.18E-50 | YP_009506665 | | |
| TFV 7 | 6887-7315 | | 142 | hypothetical protein | hypothetical protein [Common midwife toad virus] | 2.02E-86 | ASH98934 | | |
| TFV 8 | 7393-11277 | | 1294 | DNA-dependent RNA polymerase II largest subunit | DNA-dependent RNA polymerase II largest subunit [Red-eared slider ranavirus] | 0 | QJU69587 | | |
| TFV 9 | 11629-14475 | | 948 | NTPase | putative NTPase [Tiger frog virus] | 0 | QKG82681 | | |
| TFV 10 | 14491-14904 | | 137 | hypothetical protein | hypothetical protein [Tiger frog virus] | 1.76E-95 | ABB92276 | | |
| TFV 11 | 15255-15467 | | 70 | hypothetical protein | hypothetical protein FV3gorf11R [Frog virus 3] | 2.13E-42 | YP_031589 | | |
| TFV 12 | 15533-16426 | | 297 | hypothetical protein | hypothetical protein [Tiger frog virus] | 0 | QKG82684 | | |
| TFV 13 | 16625-16825 | | 66 | hypothetical protein | hypothetical protein [Tiger frog virus] | 4.00E-37 | ABB92279 | | |
| TFV 14 | 16859-17029 | | 56 | hypothetical protein | hypothetical protein [Tiger frog virus] | 2.00E-31 | QKG82686 | | |
| TFV 15 | 17136-17495 | | 119 | hypothetical protein | hypothetical protein [Tiger frog virus] | 4.70E-78 | ABB92281 | | |
| TFV 16 | 17591-18538 | | 315 | AAA-ATPase | putative A32-like virion packaging ATPase [Common midwife toad virus] | 0 | YP_009508724 | | |
| TFV 18 | 19886-21394 | | 502 | hypothetical protein | hypothetical protein [Tiger frog virus] | 0 | QKG82690 | | |
| TFV 19 | 21458-24217 | | 919 | serine/threonine protein kinase | putative 2-cysteine adaptor domain protein [Testudo hermanni ranavirus] | 0 | AJR29151 | | |
| TFV 20 | 24265-24711 | | 148 | hypothetical protein | hypothetical protein [Common midwife toad virus] | 8.80E-103 | AFA44993 | | |
| TFV 21 | 24948-25598 | | 216 | hypothetical protein | hypothetical protein [Cod iridovirus] | 2.35E-155 | ANZ57112 | | |
| TFV 22 | 25728-28655 | | 975 | D5 family NTPase/ATPase | putative D5 family NTPase/ATPase [Chinese giant salamander iridovirus] | 0 | AHA80937 | | |
| TFV 23 | 29033-30181 | | 382 | hypothetical protein | surface protein [Frog virus 3] | 0 | QDZ44761 | | |
| TFV 24 | 30584-31675 | | 363 | hypothetical protein | hypothetical protein FV3gorf24R [Frog virus 3] | 0 | YP_031602 | | |
| TFV 25 | 31878-32660 | | 260 | hypothetical protein | hypothetical protein [Rana esculenta virus] | 0 | ASQ42931 | | |
| TFV 27 | 32614-32757 | | 47 | hypothetical protein | putative eIF2-alpha like protein [Frog virus 3] | 0 | ASH99208 | | |
| TFV 28 | 32984-33763 | | 259 | eIF-2 alpha-like protein | hypothetical protein [Tiger frog virus] | 2.33E-34 | ABB92291 | | |
| TFV 29 | 33953-34141 | | 62 | hypothetical protein | putative tyrosine kinase [Frog virus 3] | 0 | QDZ44496 | | |
| TFV 30 | 34296-37208 | | 970 | tyrosine kinase | unknown [Soft-shelled turtle iridovirus] | 7.30E-116 | ACF42250 | | |
| TFV 31 | 37257-37745 | | 162 | hypothetical protein | hypothetical protein D1R28 [Bohle iridovirus] | 4.43E-61 | YP_009506689 | | |
| TFV 31.5 | 37924-38220 | | 98 | hypothetical protein | hypothetical protein PPIV_ORF81 [Pike perch iridovirus] | 1.26E-22 | ANZ56994 | | |
| TFV 32 | 38818-39237 | | 139 | hypothetical protein | hypothetical protein A190 [European catfish virus] | 1.79E-95 | YP_006347684 | | |
| TFV 33 | 39287-41212 | | 641 | neurofilament triplet H1-like protein | neurofilament triplet H1-like protein [Common midwife toad virus] | 0 | ASH97758 | | |
| TFV 34 | 41295-41486 | | 63 | hypothetical protein | unknown [Soft-shelled turtle iridovirus] | 1.00E-34 | ACF42255 | | |
| TFV35 | 41630-41959 | | 109 | hypothetical protein | hypothetical protein [Frog virus 3] | 4.49E-73 | QKO01775 | | |
| TFV37 | 42182-43075 | | 297 | hypothetical protein | surface protein [Frog virus 3] | 4.33E-77 | QDZ45983 | | |
| TFV39 | | 43484-44119 | 211 | NIF/NLI interacting factor | putative NIF/NLI interacting factor [Pike perch iridovirus] | 1.26E-152 | | ANZ56987 |  |
| TFV40 | | 44258-45955 | 565 | ribonucleotide reductase alpha subunit | class I ribonucleotide reductase [Tortoise ranavirus] | 0 | | AJR29287 |  |
| TFV41 | | 46068-46418 | 116 | hypothetical protein | hypothetical protein CH8/96_ORF52L [Testudo hermanni ranavirus] | 2.36E-72 | | AJR29135 |  |
| TFV42 | | 46506-47033 | 175 | hypothetical protein | hypothetical protein [Tiger frog virus] | 4.82E-109 | | ABB92304 |  |
| TFV44 | | 47415-50912 | 1165 | hypothetical protein | hypothetical protein [Tiger frog virus] | 0 | | QKG82715 |  |
| TFV45 | | 51454-52476 | 340 | hypothetical protein | hypothetical protein [Tiger frog virus] | 1.49E-148 | | ABB92307 |  |
| TFV46 | | 52604-53014 | 136 | hypothetical protein | hypothetical protein A190_gp105 [European catfish virus] | 1.03E-94 | | YP_006347696 |  |
| TFV47 | | 53068-53625 | 185 | hypothetical protein | RGI orf47L-like protein [Frog virus 3] | 3.10E-91 | | AHM26124 |  |
| TFV48 | | 53750-54166 | 138 | hypothetical protein | hypothetical protein [Red-eared slider ranavirus] | 1.24E-93 | | QJU69640 |  |
| TFV49 | | 54169-54420 | 83 | hypothetical protein | hypothetical protein PPIV_ORF62 [Pike perch iridovirus] | 1.91E-53 | | ANZ56978 |  |
| TFV50 | | 54528-55961 | 477 | hypothetical protein | hypothetical protein [Frog virus 3] | 0 | | QKO01790 |  |
| TFV51 | | 56221-57906 | 561 | hypothetical protein | hypothetical protein [Tiger frog virus] | 0 | | ABB92313 |  |
| TFV52 | | 58163-59230 | 355 | 3-beta-hydroxy-delta-5-C27 steroid oxidoreductase-like protein | putative 3-beta hydroxysteroid dehydrogenase [Tortoise ranavirus] | 0 | | AJR29277 |  |
| TFV53 | | 59564-61132 | 522 | myristylated membrane protein | Orf20-like protein [Tiger frog virus] | 0 | | QKG82212 |  |
| TFV54 | | 61283-61513 | 76 | nuclear calmodulin-binding protein | putative nuclear calmodulin-binding protein [Tiger frog virus] | 7.36E-49 | | QKG82213 |  |
| TFV55 | | 61551-62846 | 431 | helicase-like protein | helicase-like protein [Tiger frog virus] | 0 | | QKG82726 |  |
| TFV55.5 | | 62854-63003 | 49 | hypothetical protein | 40 kDa protein [Tiger frog virus] | 0 | | QKG82316 |  |
| TFV56 | | 63040-63444 | 134 | hypothetical protein | hypothetical protein [Tiger frog virus] | 3.08E-26 | | ABB92315 |  |
| TFV57 | | 63485-64981 | 498 | phosphotransferase | hypothetical protein [Tiger frog virus] | 6.63E-93 | | QKG82729 |  |
| TFV58 | | 64988-65119 | 43 | hypothetical protein | putative phosphotransferase [Tiger frog virus] | 0 | | QKG82730 |  |
| TFV59 | | 65423-65977 | 184 | hypothetical protein | hypothetical protein [Tiger frog virus] | 3.92E-21 | | ABB92318 |  |
| TFV60 | | 66534-67592 | 352 | hypothetical protein | hypothetical protein [Tiger frog virus] | 2.24E-128 | | ABB92319 |  |
| TFV61 | | 67752-70793 | 1013 | DNA polymerase | hypothetical protein [Tiger frog virus] | 0 | | QKG82732 |  |
| TFV62 | | 71415-75074 | 1219 | DNA-dependent RNA polymerase II second largest subunit | DNA polymerase-like protein [Tiger frog virus] | 0 | | QKG82221 |  |
| TFV64 | | 75081-75239 | 52 | hypothetical protein | DNA-dependent RNA polymerase II largest subunit [Soft-shelled turtle iridovirus] | 0 | | ACF42282 |  |
| TFV65 | | 75259-75387 | 42 | hypothetical protein | hypothetical protein D1R28_gp066 [Bohle iridovirus] | 7.71E-122 | | YP_009506724 |  |
| TFV66 | | 75453-75947 | 164 | dUTPase | putative dUTPase-like protein [Tiger frog virus] | 1.61E-113 | | QKG82225 |  |
| TFV67 | | 76080-76343 | 87 | caspase recruitment domain protein | putative interleukin-1 beta convertase precursor [Tiger frog virus] | 4.92E-50 | | QKG82737 |  |
| TFV68 | | 76561-77172 | 203 | hypothetical protein | hypothetical protein [Tiger frog virus] | 2.35E-112 | | QKG82738 |  |
| TFV69 | | 77169-77462 | 97 | hypothetical protein | hypothetical protein [Tiger frog virus] | 5.74E-60 | | QKG82739 |  |
| TFV70 | | 77517-78680 | 387 | ribonucleotide reductase beta subunit | ribonucleotide reductase small subunit diphosphate beta-like protein [Rana nigromaculata ranavirus] | 0 | | AVM86137 |  |
| TFV72 | | 79169-79324 | 51 | hypothetical protein | hypothetical protein [Tiger frog virus] | 3.48E-26 | | QKG82231 |  |
| TFV73 | | 79393-79659 | 88 | hypothetical protein | hypothetical protein [Tiger frog virus] | 9.24E-57 | | QKG82232 |  |
| TFV74 | | 79677-80051 | 124 | hypothetical protein | hypothetical protein [Tiger frog virus] | 4.20E-82 | | QKG82233 |  |
| TFV75 | | 80091-80324 | 77 | hypothetical protein | hypothetical protein [Tiger frog virus] | 3.60E-48 | | QKG82745 |  |
| TFV76 | | 80307-81101 | 264 | hypothetical protein | hypothetical protein [Common midwife toad virus] | 2.69E-115 | | AFA44939 |  |
| TFV77 | | 81495-82469 | 324 | NTPase helicase-like protein | NTPase/helicase-like protein [Tiger frog virus] | 0 | | AAL77808 |  |
| TFV78 | | 82674-83669 | 331 | hypothetical protein | hypothetical protein [Tiger frog virus] | 0 | | QKG82237 |  |
| TFV79 | | 83726-83980 | 84 | LITAF/PIG7 possible membrane associated motif in LPS-induced tumor necrosis factor alpha factor | LITAF/PIG7 possible membrane associated motif in LPS-induced tumor necrosis factor alpha factor [Lumpfish ranavirus] | 7.42E-53 | | AZY88431 |  |
| TFV80 | | 84043-84264 | 73 | hypothetical protein | hypothetical protein [Chinese giant salamander iridovirus] | 2.52E-47 | | AGK44982 |  |
| TFV81 | | 84261-84608 | 115 | hypothetical protein | hypothetical protein D1U33_gp030 [Common midwife toad virus] | 4.90E-78 | | YP_009508658 |  |
| TFV82 | | 85063-85701 | 212 | hypothetical protein | hypothetical protein [Tiger frog virus] | 9.31E-156 | | ABB92336 |  |
| TFV83 | | 85836-87554 | 572 | ATPase-dependent protease | putative ATPase-dependent protease [Tiger frog virus] | 0 | | QKG82753 |  |
| TFV84 | | 88153-89268 | 371 | ribonuclease III | ribonuclease III [Frog virus 3] | 0 | | YP_031659 |  |
| TFV85 | | 89324-89602 | 92 | transcription elongation factor-SII | transcription elongation factor-SII [Common midwife toad virus] | 9.96E-62 | | YP_009508654 |  |
| TFV86 | | 89730-90203 | 157 | hypothetical protein | hypothetical protein ToRV1_ORF22L [Tortoise ranavirus] | 8.29E-108 | | AJR29253 |  |
| TFV88 | | 90581-90868 | 95 | thymidylate synthase | cytosine DNA methyltransferase [Common midwife toad virus] | 2.37E-156 | | ASH98844 |  |
| TFV89 | | 91237-91881 | 214 | cytosine DNA methyltransferase | hypothetical protein [Tiger frog virus] | 9.24E-57 | | QKG82232 |  |
| TFV90 | | 92281-93018 | 245 | proliferating cell nuclear antige | proliferating cell nuclear antigen [Red-eared slider ranavirus] | 9.32E-180 | | QJU69601 |  |
| TFV91 | | 93093-93680 | 195 | deoxynucleoside kinase | thymidine kinase [Tiger frog virus] | 3.91E-141 | | ABB92339 |  |
| TFV92 | | 94125-94280 | 51 | hypothetical protein | hypothetical protein PPIV_ORF19 [Pike perch iridovirus] | 2.00E-20 | | ANZ56937 |  |
| TFV93 | | 94633-96429 | 598 | hypothetical protein | hypothetical protein [Frog virus 3] | 0 | | QKO01827 |  |
| TFV93 | 96462-96914 | | 150 | Erv/Alr family protein | ERV1/ALR-related protein [Bohle iridovirus] | 5.24E-108 | | YP_009506749 |  |
| TFV94 | 96982-98136 | | 384 | hypothetical protein | hypothetical protein [Tiger frog virus] | 0 | | QKG82561 |  |
| TFV95 | 98229-99620 | | 463 | major capsid protein | Major capsid protein [Tiger frog virus] | 0 | | Q91QZ8 |  |
| TFV96 | 99745-100932 | | 395 | immediate early protein ICP-46 | immediate early protein ICP-46 [Tiger frog virus] | 0 | | QKG82255 |  |
| TFV98 | 101674-101841 | | 55 | hypothetical protein | hypothetical protein ATL82 [Epizootic haematopoietic necrosis virus] | 7.85E-27 | | YP_009182011 |  |
| TFV99 | 101957-102424 | | 155 | hypothetical protein | hypothetical protein [Tiger frog virus] | 1.35E-109 | | ABB92346 |  |
| TFV100 | 102519-103613 | | 364 | DNA repair protein RAD2 | putative DNA repair protein RAD2 [Tiger frog virus] | 0 | | QKG82770 |  |
| TFV101 | 104429-105100 | | 223 | hypothetical protein | hypothetical protein D1R28_gp099 [Bohle iridovirus] | 3.53E-156 | | YP_009506757 |  |
| TFV102 | 105183-105608 | | 141 | hypothetical protein | putative myeloid cell leukemia protein [Tiger frog virus] | 2.82E-97 | | QKG82466 |  |

**Table S3** Sequence identity matrix showing the nucleotide (nt) identity of the Chad ranaviruses to 47 ranaviruses, based on the partial major capsid protein gene nt sequences (Dataset S2). Consensus sequence of Chad ranaviruses was generated based on 18 identical sequences (i.e., A-19, A-20, A-21, A-22, A-24, A-25, A-27, A-35, A-50, A-51, A-54, A-58, A-60, A-64, A-73, A17-07, A17-08, 053). See Table S1 for virus abbreviations.

**
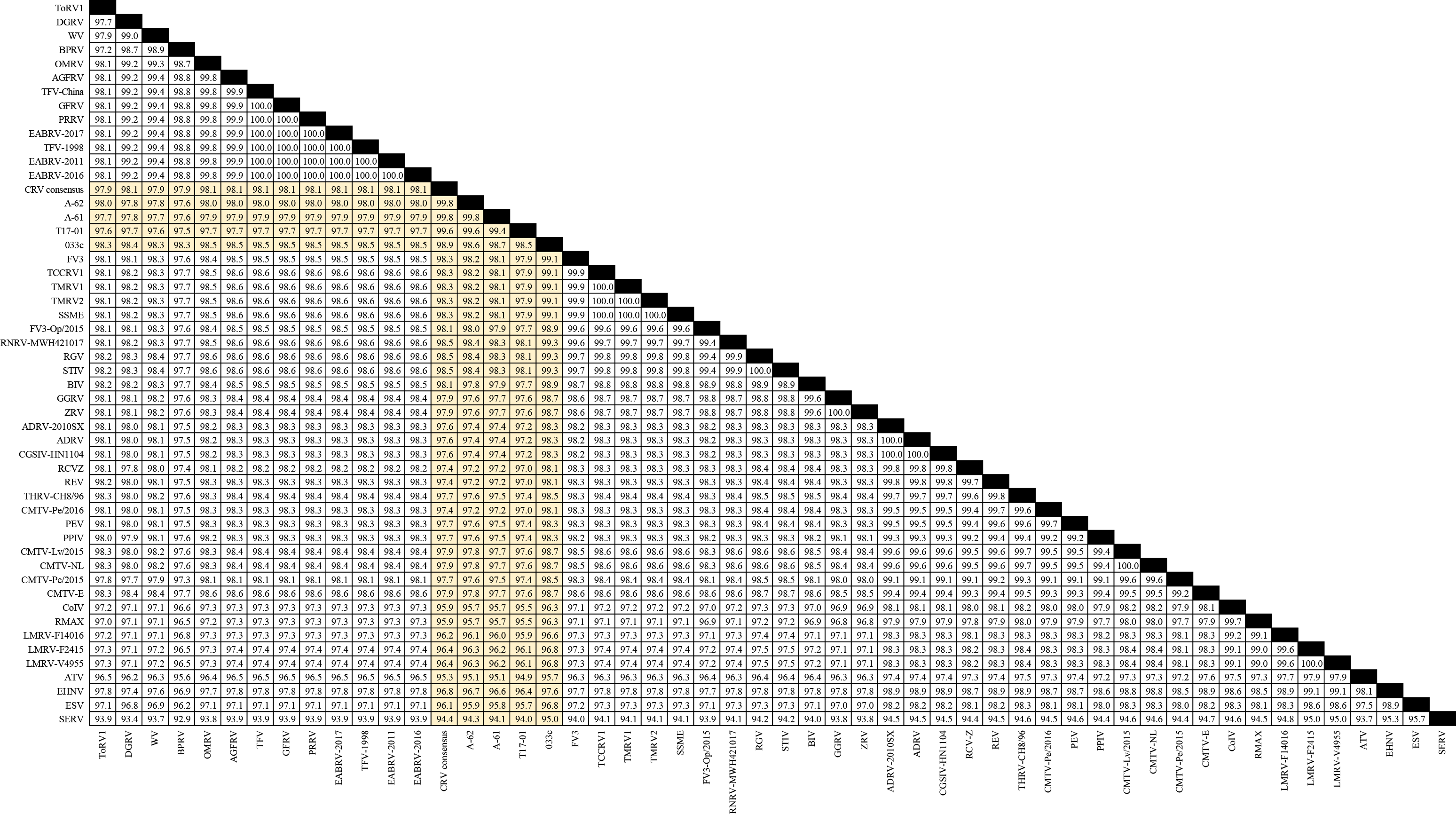
**

**Table S4** Sequence identity matrix showing the nucleotide (nt) identity of the Chad ranaviruses to 47 ranaviruses, based on the partial DNA polymerase gene nt sequences (Dataset S3). Consensus sequence of Chad ranaviruses was generated based on 9 identical sequences (i.e., A-21, A-24, A-27, A-35, A-36, A-50, A-55, A17-07, 053). See Table S1 for virus abbreviations.

**
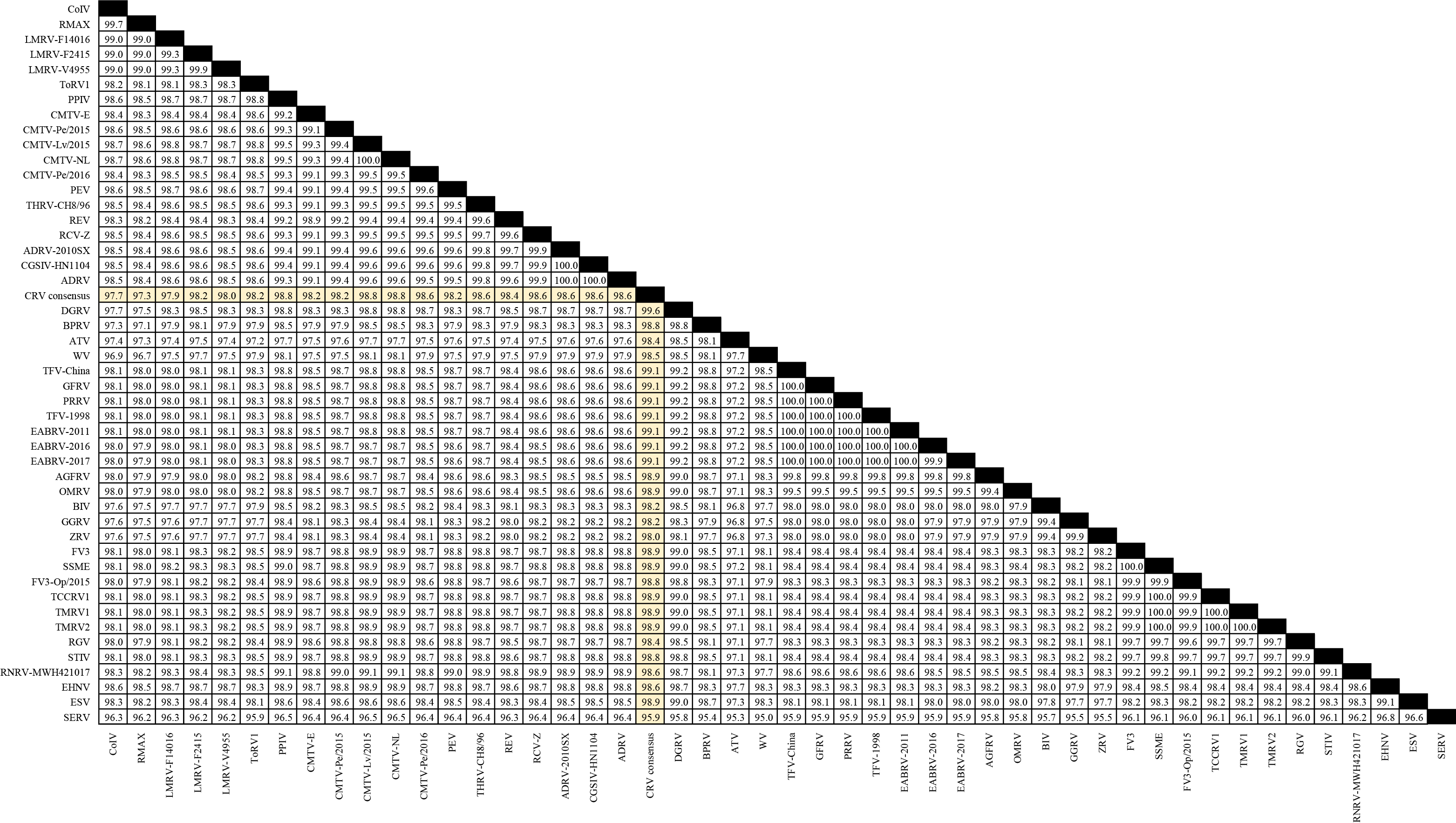
**

**Table S5** Sequence identity matrix showing the nucleotide (nt) identity of the Chad ranaviruses to 47 ranaviruses, based on the partial ribonucleotide reductase alpha gene nt sequences (Dataset S4). Consensus sequence of Chad ranaviruses was generated based on 4 identical sequences (i.e., A-21, A-50, A17-07, 053). See Table S1 for virus abbreviations.

**
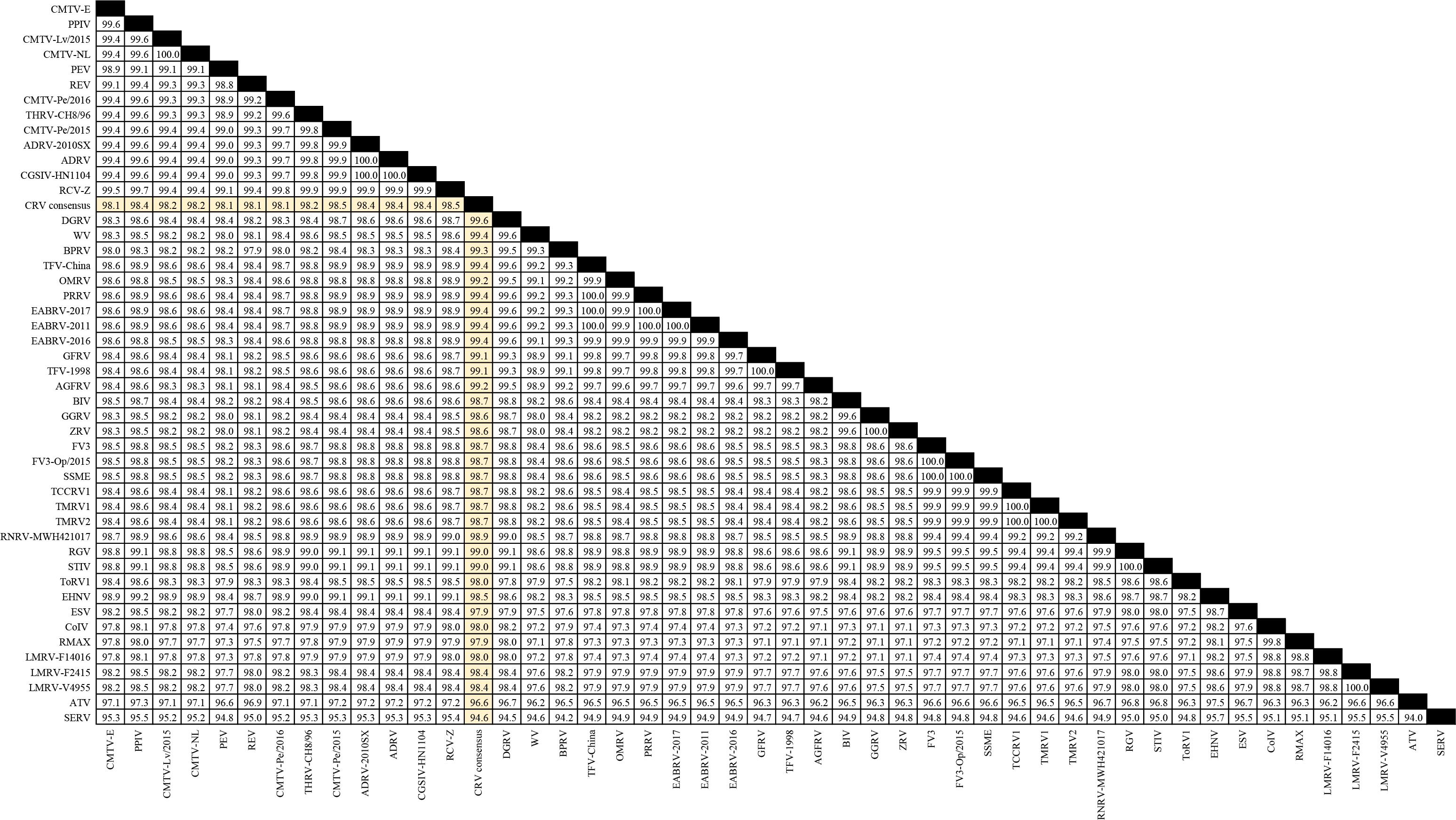
**

**Table S6** Sequence identity matrix showing the nucleotide (nt) identity of the Chad ranaviruses to 47 ranaviruses, based on the partial ribonucleotide reductase beta gene nt sequences (Dataset S5). Consensus sequence of Chad ranaviruses was generated based on 6 identical sequences (i.e., A-21, A-27, A-36, A-50, A17-07, 053). See Table S1 for virus abbreviations.

**
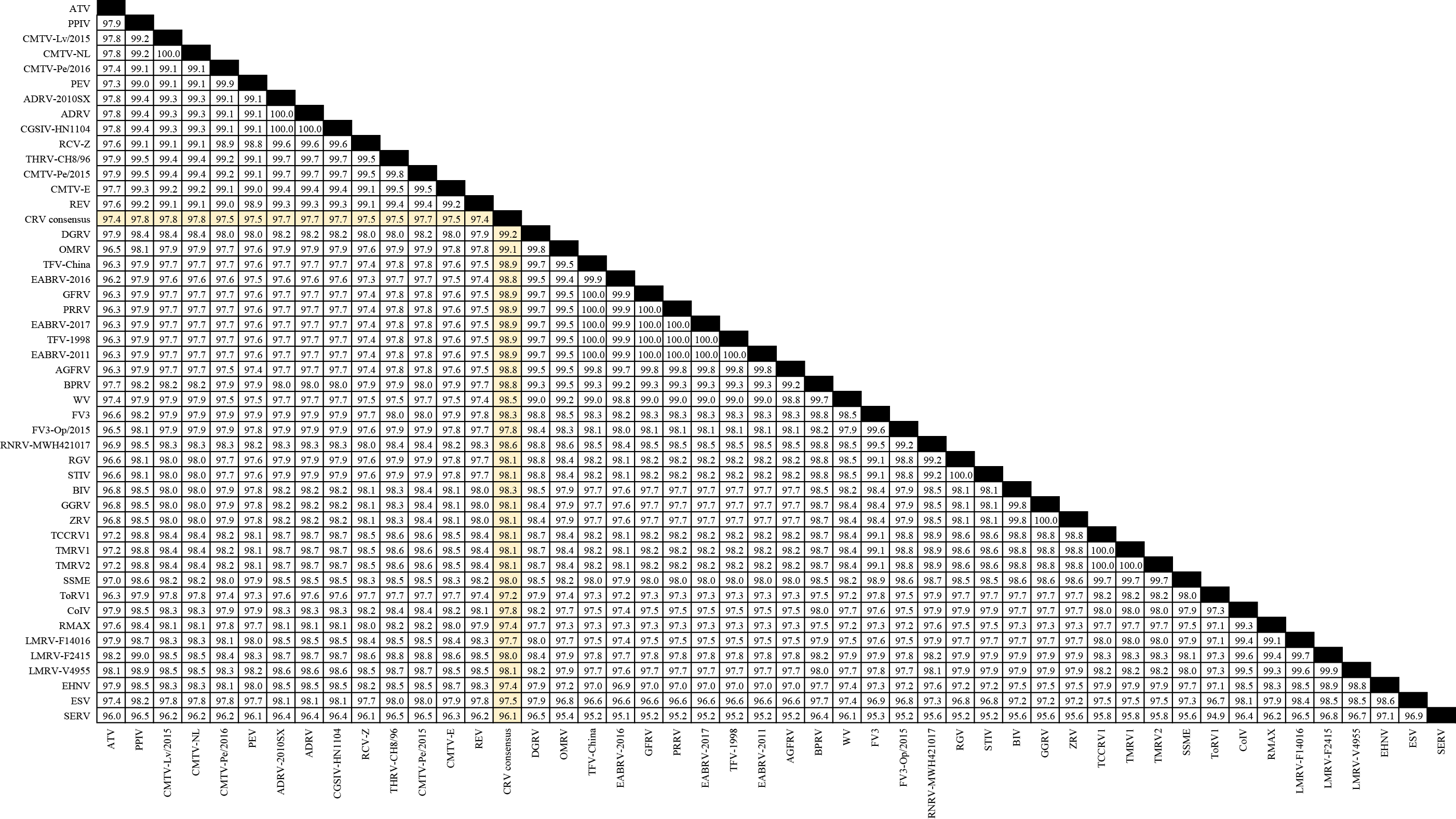
**

**
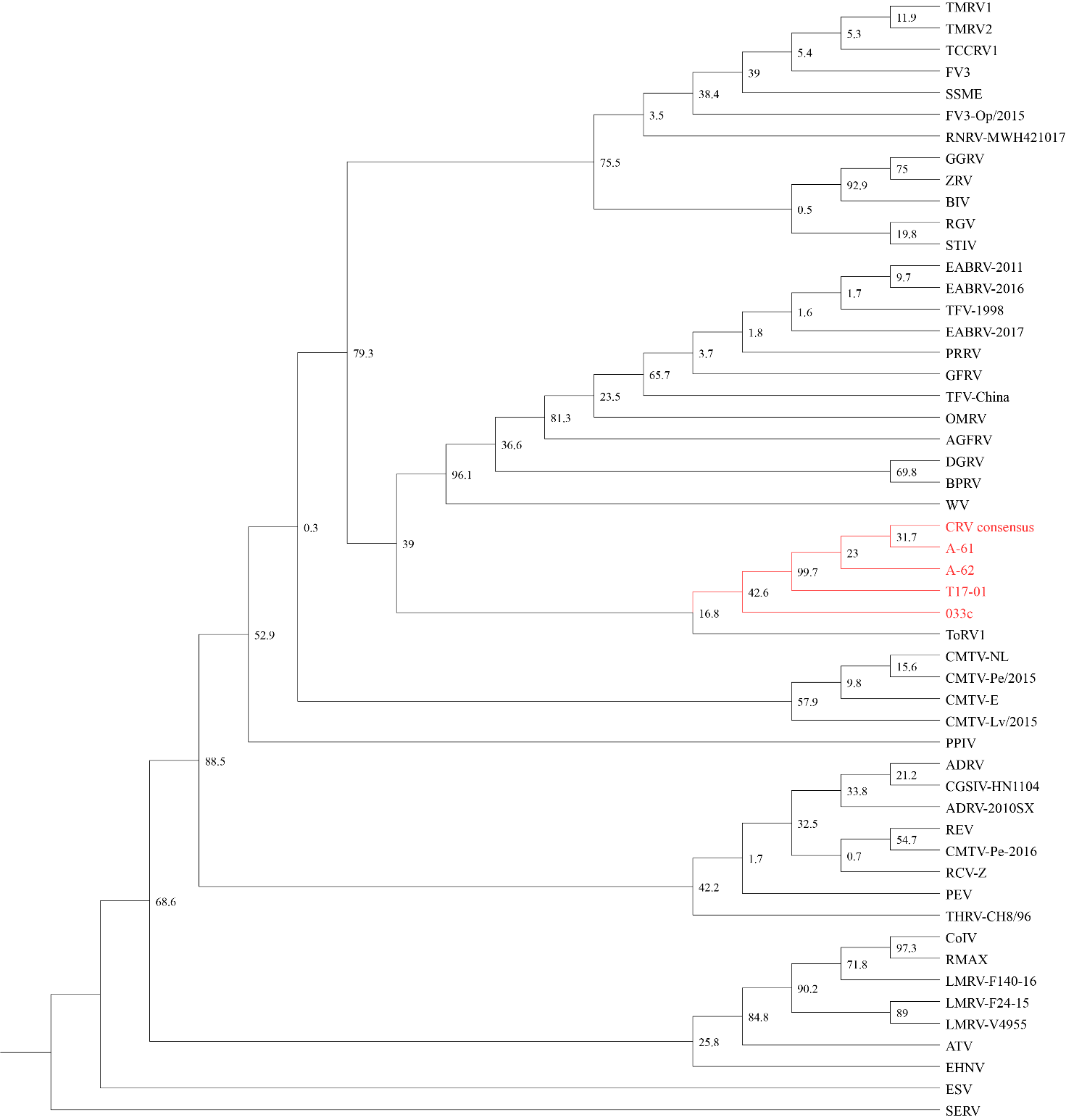
**

**Figure S1** Maximum Likelihood cladogram depicting the relationship of the Chad ranavirus sequences to 47 ranaviruses based on the partial major capsid protein gene nucleotide sequences (Dataset S2). Clade support was assessed by running 1000 bootstrap replicates with values presented at each node. The consensus sequence of the Chad ranaviruses was generated based on 18 identical sequences (i.e., A-19, A-20, A-21, A-22, A-24, A-25, A-27, A-35, A-50, A-51, A-54, A-58, A-60, A-64, A-73, A17-07, A17-08, 053). See Table S1 for virus abbreviations.

**
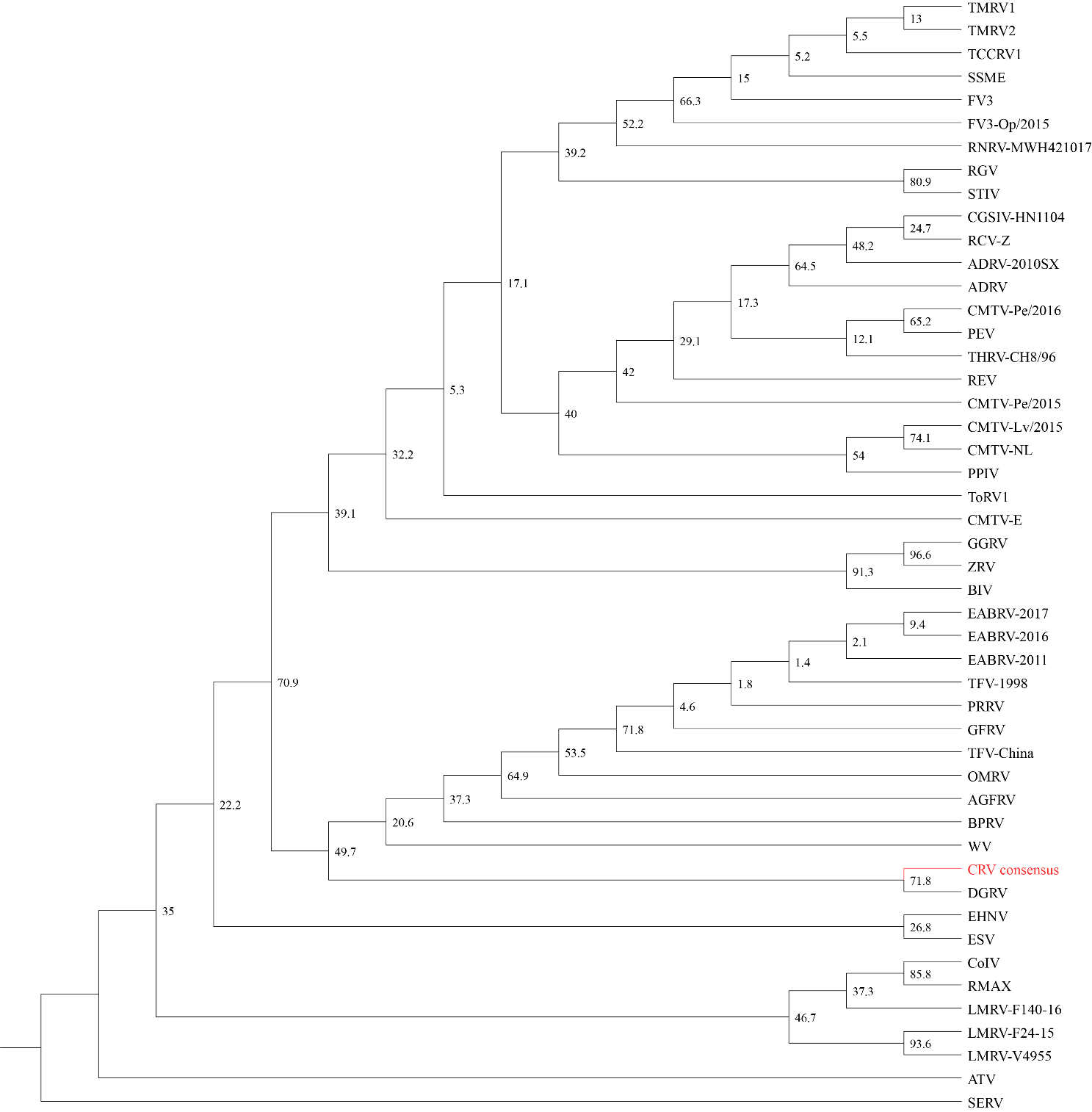
**

**Figure S2** Maximum Likelihood cladogram depicting the relationship of the Chad ranavirus sequences to 47 ranaviruses based on the partial DNA polymerase gene nucleotide sequences (Dataset S3). Clade support was assessed by running 1000 bootstrap replicates with values presented at each node. The consensus sequence of the Chad ranaviruses was generated based on 9 identical sequences (i.e., A-21, A-24, A-27, A-35, A-36, A-50, A-55, A17-07, 053). See Table S1 for virus abbreviations.

**
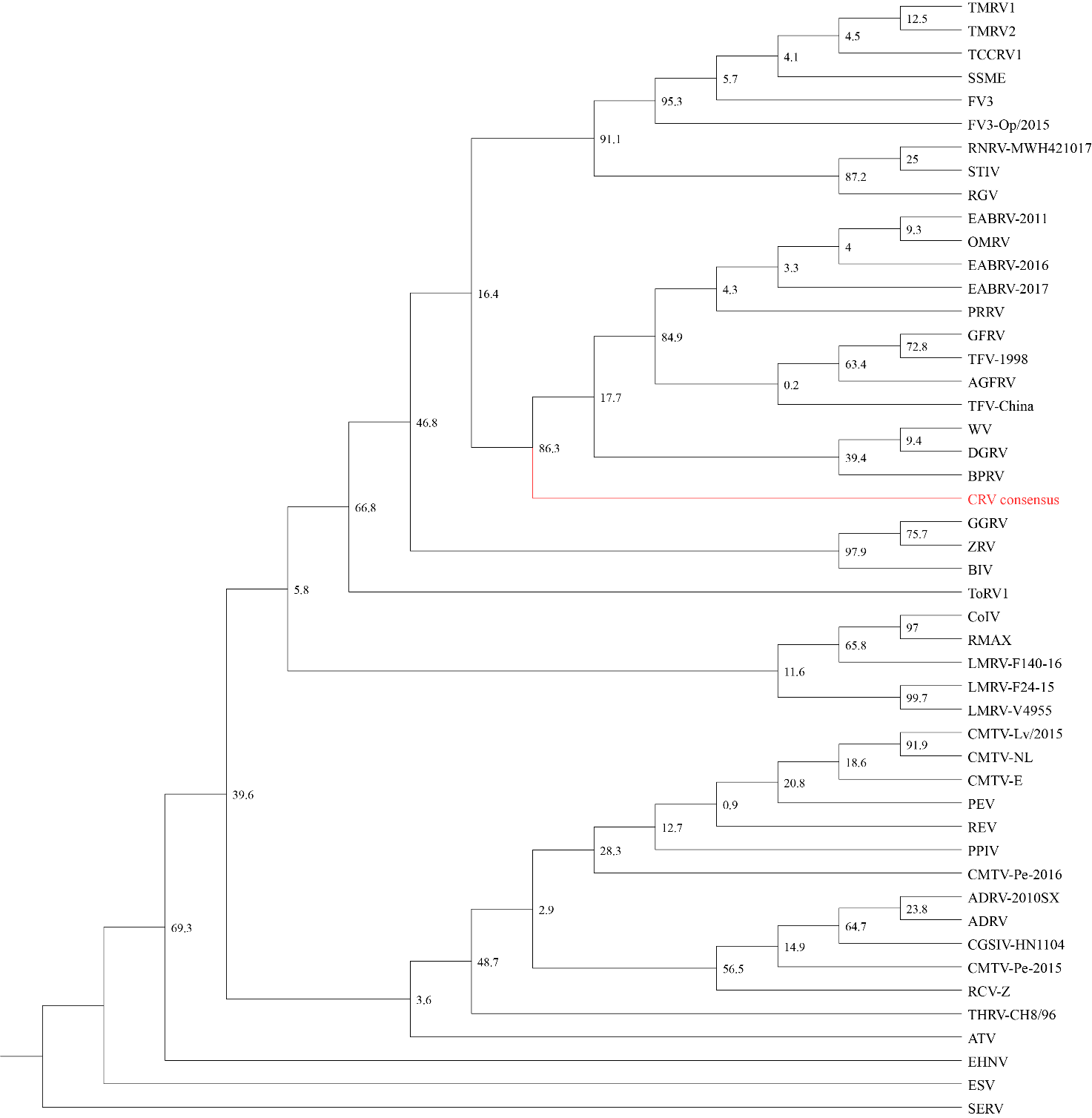
**

**Figure S3** Maximum Likelihood cladogram depicting the relationship of the Chad ranavirus sequences to 47 ranaviruses based on the partial ribonucleotide reductase alpha gene nucleotide sequences (Dataset S4). Clade support was assessed by running 1000 bootstrap replicates with values presented at each node. The consensus sequence of Chad ranaviruses was generated based on 4 identical sequences (i.e., A-21, A-50, A17-07, 053). See Table S1 for virus abbreviations.

**
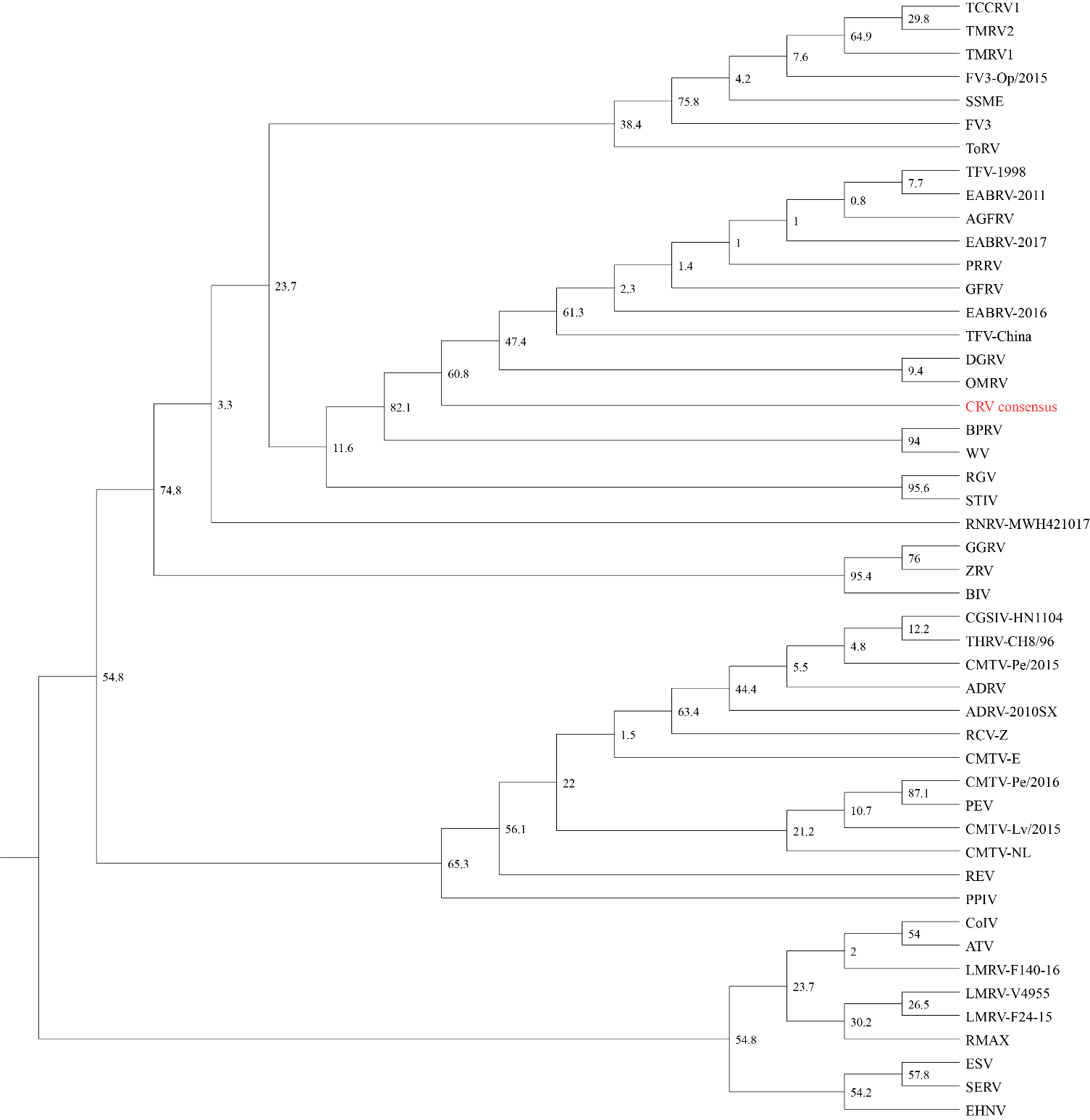
**

**Figure S4** Maximum Likelihood cladogram depicting the relationship of the Chad ranavirus sequences to 47 ranaviruses based on the partial ribonucleotide reductase beta gene nucleotide sequences (Dataset S5). Clade support was assessed by running 1000 bootstrap replicates with values presented at each node. The consensus sequence of Chad ranaviruses was generated based on 6 identical sequences (i.e., A-21, A-27, A-36, A-50, A17-07, 053). See Table S1 for virus abbreviations.

**Figure S5.** Locally Collinear Blocks (LCBs) alignments visualize genomic arrangement of the Chad ranavirus compared to other members of the genus *Ranaviruses* based on the full genome analyses. See Table S1 for virus abbreviations.
